# Glaucoma triage system: results of implementing a virtual clinic

**DOI:** 10.1007/s00417-023-06039-8

**Published:** 2023-03-30

**Authors:** Diogo Bernardo Matos, Rafael Correia Barão, Patrícia José, Afonso Cabrita, André Diogo Barata, Luís Abegão Pinto

**Affiliations:** 1 Centro Hospitalar Universitário Lisboa Norte – Hospital Santa Maria, Lisbon, Portugal; 2 Centro de Estudos das Ciências da Visão, Faculdade de Medicina da Universidade de Lisboa, Lisbon, Portugal; 3 Departamento de Oftalmologia, Hospital de Santa Maria, Av. Prof. Egas Moniz, 1649-035 Lisbon, Portugal

**Keywords:** Service delivery, Virtual clinic, Glaucoma, Model of care, Triage service

## Abstract

**Purpose:**

This study describes the virtual clinic triage system implementation process at Hospital Santa Maria—Centro Hospitalar Universitário Lisboa Norte (HSM-CHULN) and analyzes its results regarding healthcare outcomes for the patients and the system.

**Methods:**

A retrospective analysis was performed, comparing two cohorts (pre-virtual cohort; virtual triage cohort). Outcomes related to waiting time, number of hospital visits, decisions at first contact, and ancillary exam–based decisions were reported.

**Results:**

Two hundred and ninety-two charts were reviewed (pre-virtual cohort: 132; virtual cohort: 160). Mean waiting time between referral and the first medical contact with the glaucoma department decreased on average by 71.3 days (human contact: 286.6 days; virtual triage contact: 215.3 days). Triage system significantly decreased waiting time for glaucoma patients, with an average decrease of 326.8 days between referral and treatment decision. Triage staging allowed to label 107 (66.9; 95% confidence intervals (CI): 59.6%, 74.2%) as non-urgent; 30 (18.8%; 95% CI: 12.7%, 24.9%) as urgent, and 23 (14.3%; 95% CI: 8.9%, 19.7%) as immediate contact, with the scheduling of future appointments reflecting National Institute for Health and Care Excellence (NICE) guidelines in every patient. Moreover, the number of visits to perform the same exams and obtain the same clinical decisions was reduced by 63.6%.

**Conclusion:**

Our virtual screening strategy significantly decreased waiting time, number of hospital visits, and increased chances of data-assisted clinical decision. While results can be further improved, this system can add value in an overburdened healthcare system, where triage systems with remote decision-making may be valuable tools in optimizing glaucoma care, even without allocation of extra resources.


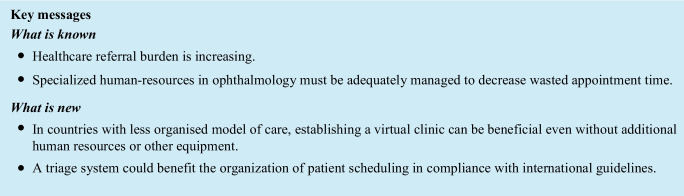


## Introduction

Higher life expectancy has increased healthcare referrals and the need for medical appointments. Current models of care for many chronic diseases pose yet another challenge, requiring many follow-up appointments to undergo tests and to evaluate disease progression. In specialized areas such as ophthalmology, human resources are scarce and modelling predicts that these problems are set to worsen if patient management remains unchanged [1].

In the current model, patient waiting time frequently surpasses the recommended periods, which is often detrimental to patient care and may impair long-term visual outcomes.

To tackle system overload, various multidisciplinary collaborations and service delivery innovations have been tested, including telemedicine and virtual clinics, which allow rapid and reliable patient assessment [2] through the efficient usage of consultant time by assuring complementary exams are being performed on schedule, and by organizing their information in a user-friendly platform to ensure rapid decision-making in compliance with up-to-date guidelines. [3] In ophthalmology, many virtual clinics have been established worldwide [4–9] to deal with the heavy burden of patient referral and follow-up appointment overload, particularly in glaucoma and retina departments. Kotecha et al. [8, 10, 11] described the development process of a virtual clinic for glaucoma patient follow-up at Moorfields Eye Hospital in London, whereas Trikha et al. [12] described using virtual clinics as a triage model for patient prioritization upon glaucoma referral.

New models of care may allow fewer patient visits [1], ensuring efficiency in healthcare resource usage while decreasing patients’ length of stay at the hospital’s facilities [13, 14]. Furthermore, there are also benefits to service providers and clinicians themselves by shifting their service towards decision-making and patient care.

In early 2019, the Ophthalmology Department at Hospital Santa Maria—Centro Hospitalar Universitário Lisboa Norte (HSM-CHULN) decided to implement a virtual clinic triage system to address the delay in first medical contact upon glaucoma department referral and to ensure signalling of urgent/surgical cases.

## Purpose

This paper describes the virtual clinic triage system implementation process at HSM-CHULN and analyzes its results regarding healthcare outcomes for the system when compared to the previous model.

## Materials and methods

### Study design

A retrospective analysis was performed, comparing two cohorts (pre-virtual cohort; virtual triage cohort).

### Setting

This study was conducted at HSM-CHULN, a government-funded teaching hospital and tertiary care nationwide referral center.

### Methodology

Data from all the patients referred to the glaucoma department of HSM-CHULN between 2018 and 2020 has been requested to the Information Management Department of HSM-CHULN. All patients who were given a first glaucoma appointment from February to September 2018 were included in group 1 (i.e., prior to virtual clinic), and all patients with a first glaucoma appointment from February to September 2019 were included in group 2 (i.e., after virtual clinic implementation). The first cohort served as a control for the cases that underwent virtual screening, as the requirements for referral were not changed throughout both groups. A case report formulary was created, and all patients were contacted and asked to sign an informed consent agreement before file opening. All collected data was anonymized in compliance with the governing General Data Protection Regulation (GDPR) European 2018 rules. All individuals under 18 years old of age were excluded from this study. This study has been approved by the ethics committee of HSM-CHULN prior to data collection and was designed and conducted in compliance with the Declaration of Helsinki for medical research.

### Data and statistics

Data collected from the hospital’s Electronic Medical Record (EMR) included variables shown in Table 1. Action points of each medical appointment were recorded using a multiple-choice menu (ie: exam rescheduling; follow-up; treatment change; surgery; discharge).

**Table 1:** Data variables collected from patient’s electronic medical record

Demographic data
Age
Gender
Clinical eye-related data
Referral motive
Therapy upon referral
Intraocular pressure upon referral
Risk stratification NICE guidelines (1)
Timing
Exams
Medical appointments
Action point
Outcome of contact
Surgical date

### Model description

A virtual glaucoma clinic triage system was implemented at HSM-CHULN to deal with all new patients referred to the department. All patients undergo a general ophthalmology appointment before being referenced for a glaucoma department appointment.

**Fig. 1:**
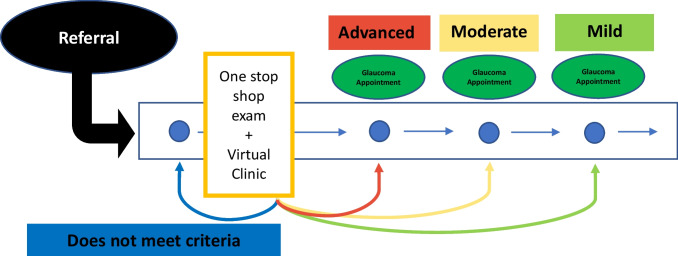
Virtual clinic patient journey model. The horizontal axis represents time; the blue circle represents patient journey

This model was designed to address the delayed primary medical contact upon referral to the glaucoma department, by ensuring a faster contact on a triage virtual appointment to tailor in-person appointment scheduling according to the patient’s needs. The virtual glaucoma clinic included all patients referred to the glaucoma department of this hospital, all of which were enrolled in a standardized protocol as depicted in Fig. 1.

Upon entrance into the hospital’s referral system to the glaucoma department, every patient was enrolled in the virtual clinic triage system and scheduled an appointment with an orthoptist technician to collect patient data according to a standard protocol including (1) therapeutic compliance questionnaire; (2) G2 dynamic automated static perimetry (Octopus® 900); (3) retinal nerve fiber layer (RNFL) and Bruch’s membrane opening minimum rim width (BMO-MRW) using a spectral-domain optical coherence tomography (SD-OCT; Heidelberg® Spectralis); (4) color fundus photography (Canon® CR-2 Plus Retinal Camera); (5) intraocular pressure (IOP) (iCare® rebound tonometry); and (6) ultrasound pachymetry (Alcon® Ocuscan RP). The orthoptist was previously certified by a glaucoma consultant in performing these exams, but all data harvested was interpreted by the glaucoma consultant.

Any patient complaints or complementary comments were also recorded by the technician and written on EMR, to be reviewed during the virtual triage appointment by the glaucoma consultant. All data was collected on the same appointment on a “one-stop shop” approach and the data introduced on the patient’s EMR—EPR Multiplatform, by Glintt®, currently in use at HSM-CHULN.

Data was subsequently reviewed post hoc by one of HSM-CHULN glaucoma consultants, who performed the virtual clinic triage at the hospital by evaluating patient information and segregating the patients into 4 different categories: (a) discharge; (b) mild disease/non-urgent; (c) moderate disease/elective; (d) severe disease/urgent; according to their disease, progression risk, and the timing for further follow-up recommendation in compliance with the National Institute for Health and Care Excellence (NICE) glaucoma guideline [15].

Upon finishing the evaluation, all patients received their prescription, a pre-filled written report stating their disease and progression risk, and were scheduled for an in-person glaucoma appointment according to patients’ clinically relevant needs and suitable timeline in between the next week up to 18 months, following NICE recommendations [15].

A conservative approach was taken and only patients who showed no signs of disease and/or presented a very low risk of developing glaucoma were directly discharged at the virtual clinic triage without the need for in-person medical consultation and were also given a written report stating this information. Upon discharge, patients would be followed as needed by the referring physicians, with the possibility of a new referral should it be deemed necessary. All data, reports, and prescriptions were directly introduced to the patient’s EMR.

The virtual glaucoma clinic began in February 2019 and has been working continuously since its debut. No additional staff was requested for this project, and it only encompassed a schedule re-organization. Neither the number of staff members nor the number of exams performed was modified, but the involvement of the orthoptist team in the development of this model of care allowed us to tailor the patient journey throughout the ancillary exam by organizing a model where each patient would undergo all the required ancillary exams consecutively, with the same orthoptist technician on the same appointment, using the one-stop shop model, instead of performing each of the ancillary exams in different appointment days, according to each ancillary exam calendar availability.

To evaluate differences between continuous, normally distributed variables before and after the virtual clinic setup, unpaired Student’s *T*-tests were used. Non-parametric tests were used for median comparison across samples, to control for non-normal data distribution whenever needed. To assess differences between categorical variables before and after the virtual clinic setup, contingency tables were drawn, and Pearson’s chi-squared tests were used to determine the magnitude of these effects.

For every statistical test used, a two-sided *p* value < 0.05 was considered statistically significant to reject the null hypothesis and 95% confidence intervals were routinely used. GraphPad Prism®8.4.2 for Windows (GraphPad Software, San Diego, CA, USA) was used to perform statistical testing and create tables and charts.

## Results

The patient sample was organized into two groups; the first group corresponds to the patients who received referral before the virtual clinic triage system was set up (02/2018–09/2020, *n* = 132), and the second group corresponds to the patients who received referral when the virtual clinic triage system was already functioning and who were enrolled in the virtual clinic standardized protocol (02/2019–09/2019, *n* = 160).

A total of 292 patients’ EMRs were reviewed, 67.1 ± 16.8 years old, 152 women, medicated on average with 1 topical hypotensive class, and presenting intraocular pressure of 19.5 ± 6.5 mmHg upon referral.

Triage staging allowed to label 107 (66.9; 95% confidence intervals (CI): 59.6%, 74.2%) as non-urgent; 30 (18.8%; 95% CI: 12.7%, 24.9%) as urgent, and 23 (14.3%; 95% CI: 8.9%, 19.7%) as immediate contact.

### Sampling comparability

The samples were found to be comparable, with no statistically significant differences between the two groups regarding gender, age, or treatment status (i.e., naïve vs. under anti-glaucomatous medication) upon referral with no statistically significant difference between the two experiment branches (with all *p* values > 0.100).

### Waiting periods

#### ***Waiting time from referral to first glaucoma department medical contact***

First medical contact was anticipated with the virtual clinic triage system, which allowed a faster screening by the glaucoma consultant and therefore contributed to a needs-based appointment scheduling.

A strong statistically significant difference was found in the number of days between referral and first medical contact (standard care: 286.6 ± 153.2 days vs. virtual clinic: 215 ± 111.5 days; *p* < 0.0001) with an average decrease of 71.3 waiting days from referral to first medical contact.

#### Waiting time to first ancillary exam–based clinical decision

The average time for the first ancillary exam–based decision before virtual clinic implementation was 553.7 ± 221.1 days, whereas after the virtual clinic setup, it averaged 226.9 ± 124.9 days. This is strongly statistically different (*p* < 0.0001) and represents an average decrease of 326.8 days.

The new model of care therefore assured a reduction of 59.1% in the waiting time between referral and the first ancillary exam–based clinical decision.

#### Decision-making and appointment action point

##### A. The up-to-datedness of complementary exams

Virtual clinic triage decisions were made resorting to ancillary exams performed 42.56 days before (on average), down from 249.6 days under the previous system (p < 0.001). Centering examinations on the patient and scheduling the post hoc analysis shortly afterwards allowed for reducing more than 6 months (≅ 180 days; 82.9%) the time between exams and their clinical assessment.

##### B. Appointment action outcomes

Action points on the first medical glaucoma contact were found to be strongly statistically different (p < 0.0001). In nearly half of the sample prior to the virtual clinic, the first glaucoma appointment was used to schedule ancillary exams. By contrast, this did not happen to any of the patients under the new system where every patient underwent the same standardized exam protocol before the first medical glaucoma contact. Perhaps a follow-up of having updated information, the number of immediate discharges at the first visit was 5 times higher in the second group. Another visible consequence is that patients in virtual clinics were twice as likely to be offered an intervention already on their first medical glaucoma contact.

### Number of hospital visits per patient

The number of hospital visits was also studied and found to have strong statistically significant differences (*p* < 0.0001) with 97.5% of patients needing a single hospital visit to conclude all the complementary exams after the implementation of the virtual clinic, against 71.21% of patients before the virtual clinic was implemented. After the virtual clinic was introduced, the organization of the complementary diagnostic procedures to be performed all on the same day as a protocol has managed to reduce the number of hospital visits by at least 20.38%.

### Implementation progress

Upon virtual clinic implementation, the previous waiting list was imported into the new system, to prevent follow-up losses. Despite putting the new structure through a stress test, this choice assured a patient-safe transition between systems while forcing the virtual glaucoma clinic to deal with the prior waiting list burden in its debut months.

The first 20 patients enrolled in the virtual clinic presented an average of 370.2 ± 53.63 days between referral and first medical contact, whereas the latest 20 patients enrolled in the virtual glaucoma clinic while we conducted this sample presented an average waiting time between referral and first medical contact of 77.15 ± 32.12 days (*p* < 0.0001).

### Missed appointments and exams

When patients miss an appointment, this creates inefficiency in the system, as this occupies a schedule without contributing to healthcare delivery, therefore increasing the waiting times of the whole system. Despite the promising results shown above on the improvement of the virtual triage one-stop shop system, 39 patients (24.37%) missed their scheduled exam day, therefore leading to a rescheduling on another date while wasting ancillary exam usage.

We have no data from the previous model to compare absenteeism rates between the two branches, but the one-stop shop model for ancillary exams (i.e., every exam is performed on the same day) has decreased the number of patient visits required to perform the same diagnostic ancillary exams, potentially contributing to avoid missed appointments due to a lower appointment burden pending over each patient.

## Discussion

### Waiting periods

#### Waiting time from referral to first glaucoma department medical contact

**Fig. 2:**
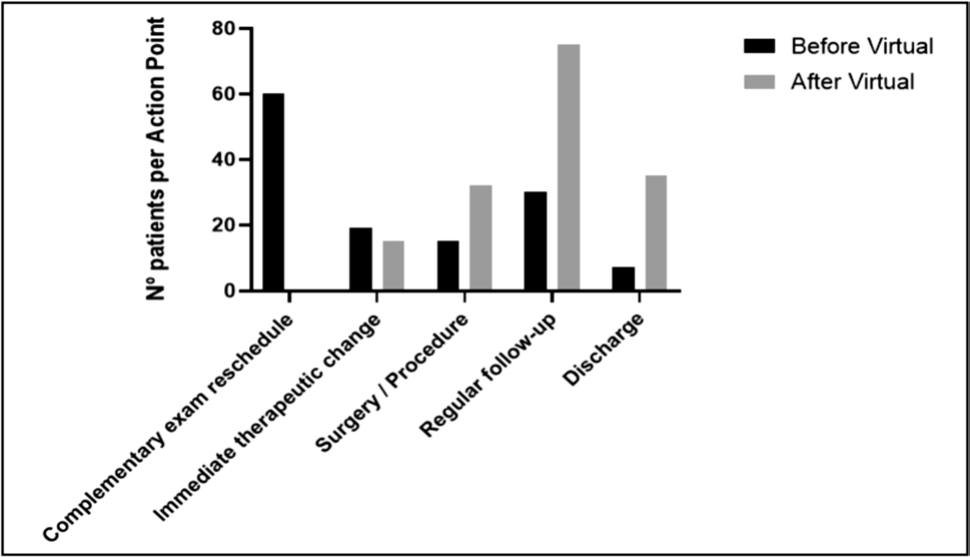
Action point of first glaucoma contact, before and after virtual glaucoma clinic. No. number

The time between referral and first glaucoma contact has been reduced to 71.3 waiting days under the new paradigm. Nevertheless, the magnitude of this reduction does not yet reflect the total efficiency improvement in the later phases of implementation, because all patients who were already waiting for the first medical contact in the previous model were enrolled in the virtual clinic referral system to avoid losing track of previously referred patients. This ensured patient safety in care access but required a bigger effort to deal with the previous waiting list before the new model of care effects were apparent (Fig. 2).

#### Waiting time to first ancillary exam–based clinical decision

The average time for the first ancillary exam–based decision decreased by an average of 326.8 days.

This occurred mostly because, in the previous model of care, patients would not be assigned any ancillary exams before glaucoma department referral, which delayed their execution and therefore precluded the glaucoma consultant from having the results on which to base clinical decisions upon the first medical glaucoma contact.

Although not every glaucoma clinical decision needs to be ancillary-based, upon virtual clinic triage system implementation, the availability of ancillary exams on which to base remote clinical decisions was of paramount significance, mainly when establishing which patients did not have glaucoma and were also at low risk of developing it, in which case the patient would be discharged without the need for an in-person appointment with a glaucoma consultant. These patients were given a written report stating they did not have glaucoma and were neither at a greater risk of developing it than the general population.

Nevertheless, glaucoma is a chronic, typically slow-progressing disease and the main benefit observed by this model was therefore not the up-to-datedness of the ancillary exams but rather their existence. Before the implementation of the virtual clinic and the one-stop shop model for complementary exams, patients would most frequently come to the first medical glaucoma appointment without any ancillary exam on which to base decision-making. By ensuring a standardized protocol of exams for every patient before medical contact, this model allowed the glaucoma consultants to avoid appointments for exam scheduling, and the number of immediate discharges at the first visit was 5 times higher after the virtual clinic was set up. Another visible consequence is that patients under virtual clinics were twice as likely to be offered an intervention already on their first glaucoma contact, and the early recognition of surgical intervention need is of critical importance in the context of a neuro-degenerative disease such as glaucoma.

As such, it is sustainable that the virtual clinic triage system changed the action point of the appointments mostly for patients without glaucoma and for patients who needed therapeutical changes (whether downgrading or upgrading treatment).

Even without additional financing or human resource spending, the virtual clinic triage system decreased on average by 326.8 waiting days between referral and the first ancillary-based glaucoma decision. This may have been significant for any unforeseen higher-risk patient, which was already unlikely, but may have been the most beneficial for the lowest-risk patients who might have had been waiting for a much longer time for a clinical discharge, with important stress-associated effects and unnecessary side effects of the usage of ocular hypotensive medication. This subanalysis was not performed.

### Number of patient visits

The number of hospital visits for multiple complementary diagnostic exams was significantly reduced by the one-stop shop protocol (at least 20.38%) and the number of appointments needed until the first informed decision was also greatly reduced (43.24%).

When accounting for both effects, the number of patient visits to perform the same exams and obtain the same clinical decision was reduced by a total of 63.6%.

Approximately 2 appointments were needed to achieve a decision before the virtual clinic (mean 1.89 appointments), while under the virtual clinic triage system, the same level of decisions was done at the first contact due to the availability of exams to support such decision (mean 1.08 appointments). When accounting for these effects, the number of patient visits to perform the same exams and obtain the same clinical decision was significantly reduced, thereby helping to avoid overcrowded waiting rooms and unnecessary hospital visits with little clinical advantage.

### Virtual clinic implementation process

When the implementation process began, there was a long list of pending glaucoma referrals. Upon virtual glaucoma clinic setup, all the pending glaucoma referrals were enrolled in the new delivery system. This structure ensured safety although at the cost of importing the burden of the previous model’s inefficiencies to the virtual clinic triage system.

The waiting times between referral and the complementary exams protocol or the virtual appointment started significantly higher and gradually decreased during the implementation effort, until the initial waiting list was eventually overcome with the new functioning model.

Despite this, the virtual glaucoma clinic turned up to be a suitable solution for the problem, managing to decrease the time between referral to first medical contact by almost 80% (from 370.2 ± 53.63 days to 77.15 ± 32.12 days) when adjusted for the implementation process.

### Limitation and possible way forward

Virtual clinics are not intended to replace human face-to-face contact. Instead, their key role in our model was to ensure this triage contact is faster, with an organized segregation of patients according to their needs for in-person appointment timelines, thereby using specialized human resource time in a rationalized approach and avoiding redundant appointments to achieve the same clinical decision. This healthcare model requires multidisciplinary work and a continuous team effort, to assure the patient receives the best care throughout the whole process.

The ability to detect the progression of glaucoma is not threatened while using this triage system, for every patient at risk of developing glaucomatous damage will undergo regular follow-up face-to-face appointments. To avoid setbacks from unforeseen situations, there should exist a mechanism to guarantee the patient is able to communicate complaints or clarify any doubts in a timely manner with the physician, even during the triage entrance on the system, and this was addressed in our model through an “open text” box on our therapeutic compliance questionnaire that was applied by the orthoptist technician during the one-stop shop moment of ancillary exams.

Furthermore, this model of care does not allow for an ab initio assessment of the anterior chamber periphery, iris characteristics, or the presence of other risk factors for glaucoma such as pseudoexfoliation material deposition, whose evaluation was only achieved upon the first medical glaucoma contact or when the referring doctor had already performed this evaluation and registered the observations in the patient’s EMR prior to glaucoma department referral.

## Conclusions

Health challenges are rapidly evolving with life expectancy’s steady increase, which is transforming healthcare settings to adapt to the striking demands of an ageing population. Model predictions allow us to foresee an escalation of this problem if systematic changes are not undertaken. In our study, the virtual clinic triage system has proven to be a powerful tool to significantly reduce the waiting time upon referral until the first medical contact, allowing for faster separation of patients according to severity groups, in line with NICE guidelines. Ancillary-based decisions were also quicker and enabled the glaucoma consultant to change therapeutic decisions twice as much as it did before such information was available. The discharge rate was also statistically significantly increased, which ultimately also contributed to unburdening a backlogged referral system.

In our experience, despite not having the much-needed additional human and logistical resources to address the complementary exam waiting delay, the adoption of a virtual clinic triage system into our institution has proven to be quite beneficial to reduce waiting times until the first medical contact, eliminate redundancies, and ensure faster ancillary-based clinical decisions.

All patients were still given appointments in compliance with NICE guidelines and according to their clinical needs. Unnecessary visits were avoided, but follow-up was assured through long-term scheduled visits with a standard exam protocol before the glaucoma appointment.

Overall, the virtual clinic triage system allowed a significant reduction in the waiting time until the first medical contact, and assured a faster triage of patients, thereby not relying solely on the diligence of the referral doctor for signalling an urgent patient. While doing this, the discharge rate on the first appointment has increased fivefold, sparing glaucoma consultant’s time for the most severe patients but also avoiding wasteful usage of patient’s time, preventing unnecessary patient stress, and precluding them from undergoing needless therapies with possible side effects.

## Data Availability

Data availability was ensured by online submission to a permanent data set, available at 10.6084/m9.figshare.19610007.

## Declarations

### Employment statement

None of the authors is currently employed by any institution who will benefit from this research, nor any of the authors intends to begin such relationship with any such institution.

This submission has not been published elsewhere previously and it is not being considered for any other publication simultaneously.
